# Retinal Optical Coherence Tomography-Angiography and Longitudinal Changes in Cognition and Cerebral Small Vessel Disease

**DOI:** 10.1212/WNL.0000000000218502

**Published:** 2026-09-15

**Authors:** Samuel Gibbon, Ylenia Giarratano, Charlene Hamid, Carmen Arteaga-Reyes, Una Clancy, Stewart Wiseman, Daniela Jaime Garcia, Ellen V. Backhouse, Maria Del C. Valdés Hernández, Michael S. Stringer, Michael J. Thrippleton, Francesca M. Chappell, Fergus Doubal, Ian Maccormick, Charlotte Jardine, Iona Hamilton, Gayle Barclay, Donna McIntyre, Borja Marin, Oskar Panek, Baljean Dhillon, Thomas J. MacGillivray, Miguel O. Bernabeu, Joanna M. Wardlaw

**Affiliations:** 1 Centre for Medical Informatics, Usher Institute, The University of Edinburgh, United Kingdom;; 2 Institute for Neuroscience and Cardiovascular Research, The University of Edinburgh, United Kingdom;; 3 Edinburgh Imaging Facilities, Edinburgh Imaging, University of Edinburgh, United Kingdom;; 4 UK Dementia Research Institute, University of Edinburgh, United Kingdom;; 5 Robert O Curle Ophthalmology Suite, Institute for Regeneration and Repair, University of Edinburgh, United Kingdom;; 6 Institute for Adaptive and Neural Computation, University of Edinburgh, United Kingdom;; 7 Edinburgh Medical School, The University of Edinburgh, United Kingdom; and; 8 Ophthalmology, Princess Alexandra Eye Pavilion, Edinburgh, United Kingdom.

## Abstract

**Background and Objectives:**

Cross-sectional studies suggest that retinal microvascular abnormalities measured by optical coherence tomography angiography (OCTA) are associated with cerebral small vessel disease (cSVD) and cognitive impairment, but longitudinal relationships remain unclear. We investigated whether OCTA measures are associated with brain cSVD markers and cognition over time and whether baseline OCTA was associated with brain and cognitive outcomes after mild ischemic stroke.

**Methods:**

We conducted a prospective longitudinal cohort study recruiting patients with lacunar or mild cortical ischemic stroke (modified Rankin Scale ≤2) from a hospital stroke service. Participants underwent OCTA, brain MRI, and medical and cognitive assessment (MoCA) at baseline and 1 year. OCTA metrics included vessel density (VD), foveal avascular zone area, vessel radius, tortuosity, and flow index from superficial and deep vascular layers. MRI outcomes included white matter hyperintensity (WMH) volume, basal ganglia perivascular space (PVS) volume, and mean diffusivity in normal-appearing white matter. We used linear mixed-effects models to assess (1) whether retinal measures were associated with brain and cognitive markers over time and (2) whether baseline retinal measures were associated with 1-year outcomes, adjusting for age, sex, systolic blood pressure, smoking, diabetes, stroke severity, disability, and stroke subtype.

**Results:**

A total of 189 participants (mean age 64.8 years; 37% female) were assessed at baseline and 154 at 1 year. Longitudinal reductions in retinal VD were associated with WMH increase (superficial layer, standardized β [95% CI] −0.045 [−0.067 to −0.023]; deep layer −0.050 [−0.074 to −0.026]). Increases in retinal VD and reductions in retinal flow were associated with increased PVS volume. Lower MoCA over 1 year tracked with decreases in deep retinal VD (0.136 [0.036–0.236]). Baseline retinal measures were associated with future outcomes: In particular, lower baseline VD in both layers was associated with worse MoCA at 1 year (superficial layer 0.125 [0.043–0.207]; deep layer 0.242 [0.162–0.322]).

**Discussion:**

OCTA-measured retinal microvascular features are associated with cSVD progression and worsening cognition after mild stroke, while baseline OCTA was associated with future brain and cognitive outcomes. These findings suggest that OCTA holds promise as a noninvasive tool for monitoring retinal microvascular pathology in stroke populations enriched for cSVD, warranting further investigation in larger cohorts.

## Introduction

Cerebral small vessel disease (cSVD) is a common, microvascular disorder that causes lacunar stroke and is a major cause of vascular cognitive impairment and dementia. MRI visible lesions such as white matter hyperintensities (WMHs) and perivascular spaces (PVSs) reflect structural damage because of abnormal small penetrating arterioles, capillaries, and venules.^1-3^ In addition to structural markers, quantitative techniques such as diffusion MRI can measure the movement of water molecules (mean diffusivity), which is often elevated in cSVD.^4^ However, the relatively low temporal resolution (submillimeter) of conventional MRI means that it cannot readily visualize changes in the cerebral microcirculation in vivo.

The retinal microvasculature offers an accessible window onto cerebral small vessel health: The retina and brain share developmental origins and have similar microvascular architecture.^5^ Optical coherence tomography angiography (OCTA) noninvasively images retinal capillary networks in a layer-specific manner and can generate near-histologic resolution images (∼5 μm) of vessel morphology and perfusion without contrast injection.^6^ From these images, a range of quantitative metrics can be computed (for example, vessel and capillary density, layer-specific perfusion indices, foveal avascular zone (FAZ), and tortuosity) that aim to capture structural and functional properties of the retinal microcirculation.^7,8^ Owing to the high-resolution and patient-friendly nature of OCTA, there has been great hope that the technology could enable early microvascular pathologic changes relevant to those in the brain to be detected and monitored in vivo.^9-12^

Cross-sectional OCTA studies consistently show reduced vessel density (VD) in cSVD compared with controls,^13-16^ with lower values associated with greater WMH burden (visual ratings, volume),^17-21^ more PVS,^15,22^ worse cognitive function,^23-26^ and higher white matter mean diffusivity.^27^ Lower vessel skeleton density and higher vessel diameter index correlate with larger WMH volume,^21^ and reduced capillary density has been linked to greater WMH burden (WMH burden defined in this cited article as high/low [Fazekas] or volume.)^17^ and to subcortical vascular cognitive impairment.^16^ Smaller FAZ area has been associated with greater WMH and PVS burden.^18,21^ Finally, sparser inner retinal microvasculature has been observed after lacunar ischemic stroke because of cSVD.^28^ Collectively, these cross-sectional studies suggest that retinal changes may, to some extent, parallel cerebral microvessel pathology and resulting visible lesions. However, critical questions remain about whether temporal changes in the retinal microvasculature relate to concurrent progression of cSVD and cognitive decline.

To address this gap, we assessed longitudinal (1 year) retinal OCTA, brain, and cognitive changes in a prospective cohort of patients with lacunar ischemic stroke or mild cortical ischemic stroke (controls) enriched for cSVD, the Mild Stroke Study 3 (MSS3).^29^ We addressed 2 research questions. First, are retinal OCTA features associated with brain and cognitive markers over time? Second, are baseline retinal features associated with subsequent changes in brain structure and cognitive performance?

## Methods

### Participants

The Mild Stroke Study 3 has been described in detail previously.^29^ In brief, we recruited patients from a hospital stroke service presenting with either a lacunar stroke (assuming underlying cSVD) or a minor nonlacunar cortical ischemic stroke, expected to be nondisabling (modified Rankin Scale [mRS] ≤2). Nonlacunar strokes included large artery atherothromboembolic, cardioembolic, and cryptogenic mechanisms. Stroke diagnoses were made by an expert panel through consensus, based on a review of symptoms and clinical signs, supported by diagnostic brain MRI or CT, as previously described.^29,30^ Clinical stroke subtype (lacunar or cortical) was determined according to the Oxfordshire Community Stroke Project clinical classification system, supported by brain imaging. Owing to a study requirement to assess cerebrovascular function, individuals with severe cardiorespiratory or renal failure were excluded. Baseline research assessment took place between 1 and 3 months after the index stroke and included MRI, retinal imaging, medical history and examination including vascular risk factors, and cognitive assessments. All participants were invited for follow-up assessments at 6 and 12 months, which included structural MRI, retinal imaging, and medical and cognitive assessment (MoCA).^29^

In this study, we used the full recruited population and 1-year follow-up. This is in contrast with our previous OCTA study in the same data set which used a subset of patients at baseline only (N = 123).^27^

### MRI

MRI for this study has been described in detail previously.^29,31^ All baseline and follow-up, MRI was performed on the same 3T Siemens Prisma with identical sequences, including 3D T1w, T2w, FLAIR, susceptibility-weighted imaging/susceptibility-weighted acquisition for neurovascular imaging/gradient recalled echo, and single-shell or multishell diffusion imaging. MRI data were visually assessed for index and old infarcts, WMH, lacunes, PVS, microbleeds, brain atrophy according to the STRIVE (STandards for Reporting Vascular changes on Euroimaging) critera^1^ and computationally analyzed to assess WMH volume, PVS volume in the basal ganglia, and intracranial volume (ICV) using validated methods. Using diffusion tensor imaging, we computed mean diffusivity in the normal appearing white matter. The acquisition protocol and analysis have been described previously.^29,31,32^

### OCTA Image Acquisition

**Figure 1:**
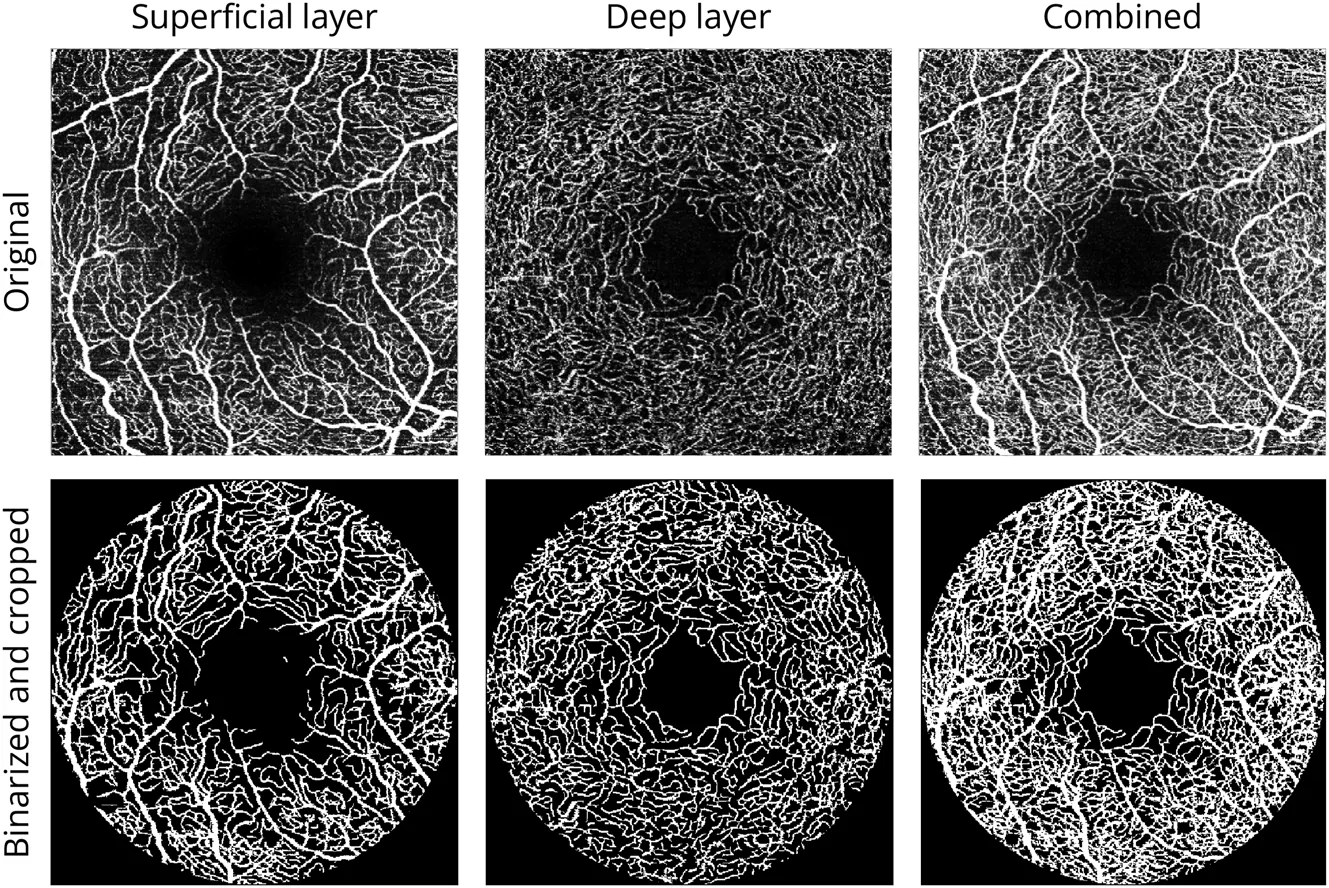
OCTA Images and Their Computationally Binarized and Cropped (Parafoveal Ring) Counterparts The summed image (right) was used to compute the foveal avascular zone (FAZ). OCTA = optical coherence tomography angiography.

Retinal imaging was performed using the SPECTRALIS platform (Heidelberg Engineering, Germany), capturing both eyes where possible. Infrared scanning laser ophthalmoscopy in the instrument localized the macula, followed by a 10 × 10° structural optical coherence tomography scan with corresponding OCTA flow data, detecting red blood cell movement. The MSS3 scan sequence included 512 B-scans at 6 μm intervals, covering a 3 × 3 mm area centered on the fovea. Two 2D images were generated covering both the superficial and deep layers (Figure 1).

### OCTA Measurements

OCTA images were processed using previously validated software OCTARIMA (OCTA Retinal Image Measurement Analysis).^7,33^ The following measurements were all obtained from within the parafoveal ring.

1. VD: proportion of the imaged area occupied by blood vessels, measured in both layers; reflects overall microvascular integrity.
2. FAZ: size of the central capillary-free zone, calculated on the combined image; larger FAZ may indicate microvascular rarefaction or ischemia.
3. Mean radius: average caliber of retinal vessels, measured in the superficial layer; changes may reflect vascular remodeling.
4. Mean tortuosity: average degree of vessel winding along their path, measured in the superficial layer; increased tortuosity can signal microvascular stress or pathology.
5. Flow index: mean OCTA decorrelation signal, measured in both layers; reflects relative blood flow.

### OCTA Quality Control

All OCTA scans underwent quality control by visual inspection of the superficial images. Each scan was evaluated according to the following 4 criteria: (1) absence of major artefacts, (2) visibility of large vessels (no blurring or “fuzziness”), (3) visibility of capillaries (no blurring in ≥80% of the scan), and (4) signal intensity (no substantial signal loss).

Each criterion was scored as 0 (does not meet) or 1 (meets criterion), yielding a total score from 0 to 4. Total scores were categorized as: reject (0–1), acceptable (2), good (3), or excellent (4). Quality assessments were performed in a masked manner by authors C. Hamid, Y. Giarratano, and S. Gibbon.

### Statistical Analysis and Covariates

WMH volume was normalized to ICV (%) and log-transformed following addition of a constant (1) to accommodate zero values. Measurements in the left eye were similar to those in the right eye. Therefore, we used linear mixed-effects models including both eyes to examine associations between retinal measures and brain/cognitive outcomes. For Research Question 1 (RQ1; is retina associated with brain/cognition over time?), models included the retinal measure, visit (baseline or follow-up), and their interaction as fixed effects, with the outcome variable measured at both time points. This interaction term tested whether the association between retinal measures and brain/cognitive outcomes differed across visits. For Research Question 2 (RQ2; is baseline retina associated with brain and cognitive outcomes?), we used baseline-adjusted models (brain at follow-up ∼ retina at baseline + brain at baseline). For both research questions, we constructed univariate and multivariable-adjusted models, the latter adjusted for baseline age, sex, baseline systolic blood pressure, smoking status, diabetes, baseline NIH Stroke Scale (NIHSS), baseline mRS, and stroke type (lacunar, cortical) and included a random intercept for participant to account for repeated measures and the nested structure of eyes within participants. A random intercept structure was used with an identity covariance matrix for the random effects. Random slopes were not included, given the limited number of time points per participant. For clarity, we provide pseudo-R code here.

RQ1: lmer(brain ∼ retina * visit + covariates + [1|subject]).
RQ2: lmer(followup_brain ∼ baseline_retina + baseline_brain + covariates + [1|subject]).

For testing cross-sectional baseline associations, we constructed linear models separately for left and right eyes, using only 1 eye per participant in each model. All continuous variables were standardized to have mean zero and unit variance; β coefficients represent effect sizes in SD units and are directly comparable across outcomes. Analyses were performed in R (version 4.4.3) using the lme4 and lmerTest packages.

Given the exploratory nature of this study and the number of retinal-brain/cognition associations tested, no correction for multiple comparisons was applied; *p* values are presented to aid interpretation but should be considered in this context.

### Standard Protocol Approvals, Registrations, and Patient Consents

The MSS3 study was approved by the South East Scotland Research Ethics Committee (18/SS/0044), and all participants gave written informed consent. This study was conducted in accordance with the Declaration of Helsinki.

### Data Availability

Data are available upon reasonable request from the MSS3 team. The OCTARIMA software is available upon request from author M.O. Bernabeu.

## Results

**Figure 2:**
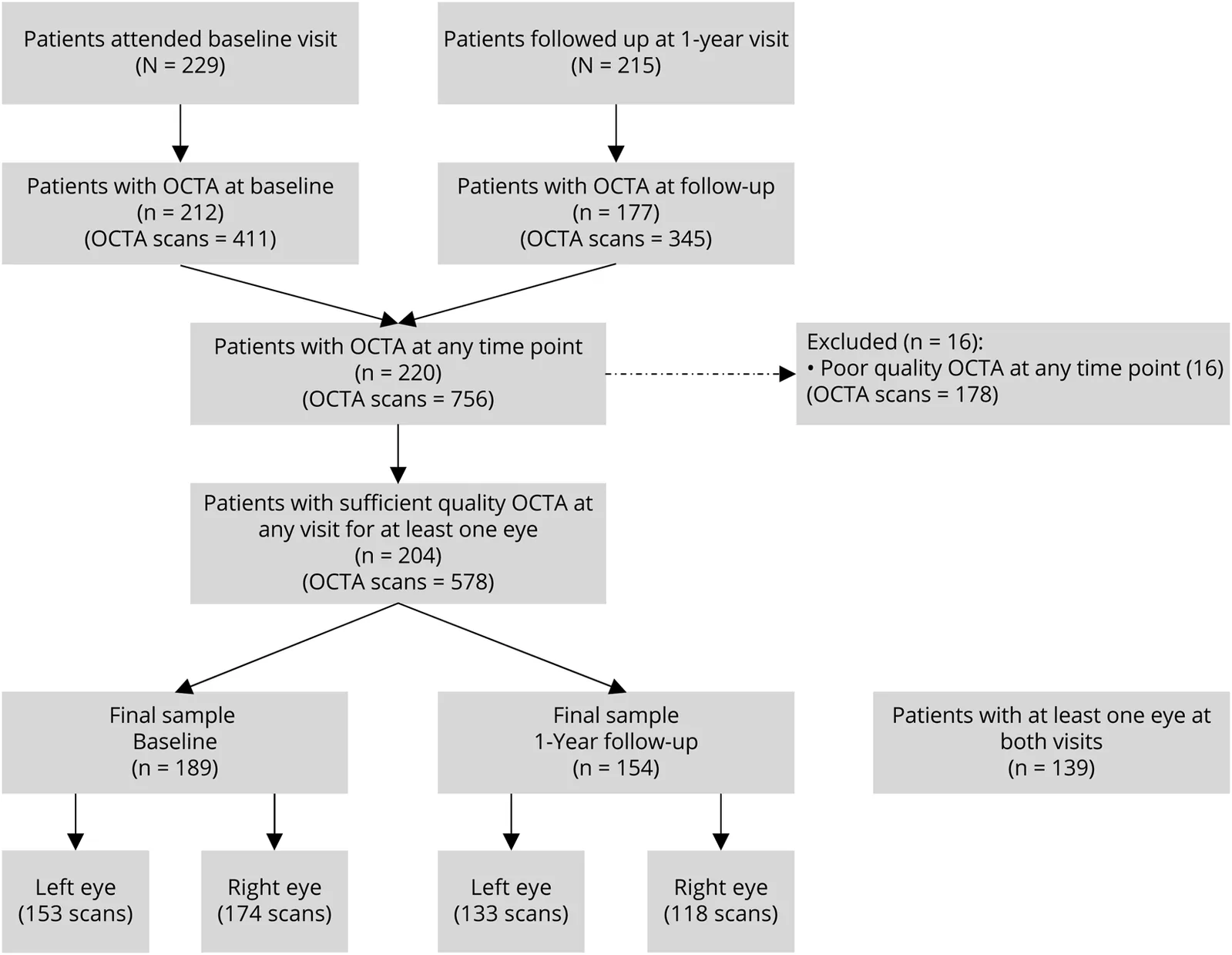
Sample Derivation Flowchart OCTA = optical coherence tomography angiography.

The final sample included 189 patients at baseline and 154 at the 1-year follow-up. Of these, 139 patients had at least 1 sufficient quality OCTA scan (either eye) at both visits (Figure 2). To improve estimate stability, mixed-effects models included all participants, even those with data from only 1 time point, and used data from both eyes where available.

At baseline, participants had a mean age of 64.8 years (SD 10.8), with 70 (37%) being female (Table 1). At the 1-year follow-up, those with available data had a mean age of 63.9 years (SD 11.3), and 48 (31.2%) were female. Between baseline and follow-up, the mean PVS volume decreased by over 400 mm^3^ [95% CI 79.9–755.0], and the median MoCA score improved by 0.8 points [95% CI –1.62 to −0.09]. Descriptively, no other net differences were observed.

**Table 1:** Patient Demographics, Covariates, Brain, Cognition, and Retinal Measures at Baseline and 1 Year

	Visit		*p* Value
	Baseline	1-y follow-up	
N (patients)	189	154	
N (OCTA scans)	327 (right eye = 174)	251 (right eye = 118)	
Covariates			
Age at baseline	64.8 (10.8)	63.9 (11.3)	0.475
Sex (female), (%)	70 (37.0)	48 (31.2)	0.306
Systolic BP at baseline	149 (21.5)	148 (19.2)	0.428
Smoking, (%)			
Never	81 (42.9)	63 (40.9)	
Current/ex	108 (57.1)	91 (59.1)	0.8
Diabetes	38 (20.1)	31 (20.1)	1
NIHSS at baseline	1.32 (1.38)	1.33 (1.40)	0.851
mRS at baseline	0.97 (0.66)	0.94 (0.67)	0.658
Stroke type (lacunar), (%)	106 (56.1)	86 (55.8)	1
Missing, (%)	1 (0.5)		
Brain measures and cognition			
Mean diffusivity in NAWM	0.76 (0.023)	0.76 (0.025)	0.227
Missing, (%)	4 (2.1)	6 (3.9)	
PVS Volume (mm^3^)	3,050 (1730)	2,630 (1330)	0.016^a^
Missing, (%)	2 (1.1)	23 (14.9)	
WMH volume	0.51 (0.41)	0.53 (0.45)	0.663
Missing, (%)	2 (1.1)	5 (3.2)	
MoCA	25.3 (3.34)	26.1 (3.55)	0.027^a^
Missing, (%)	10 (5.3)	11 (7.1)	
Retinal measures			
Total vascular density (superficial)	0.28 (0.05)	0.28 (0.05)	0.77
Total vascular density (deep)	0.27 (0.04)	0.27 (0.04)	0.544
Foveal avascular zone (μm^2^)	262000 (126000)	264000 (126000)	0.857
Radius (µm)	12.0 (1.40)	11.8 (1.42)	0.268
Tortuosity	1.07 (0.06)	1.08 (0.11)	0.497
Flow index (superficial)	4.16 (0.56)	4.10 (0.51)	0.164
Flow index (deep)	3.70 (0.34)	3.71 (0.35)	0.582

Abbreviations: BP = blood pressure; MoCA = Montreal Cognitive Assessment; mRS = modified Rankin Score; NAWM = normal appearing white matter; NIHSS = NIH Stroke Scale; OCTA = optical coherence tomography angiography; PVS = perivascular space; WMHs = white matter hyperintensities.

All values are N (%) or mean (SD).

a *p* value < 0.05.

### Baseline Associations

At baseline, lower superficial VD was consistently associated with higher WMH volume, greater PVS volume in the basal ganglia, higher mean diffusivity in normal appearing white matter (NAWM), and lower MoCA scores (eFigure 1 for all univariate and fully adjusted models). Associations with FAZ, tortuosity, radius, and flow were weak or absent. Most baseline associations attenuated after adjustment for vascular risk factors.

### Research Question 1: Is Retina Associated With Brain/Cognition Over Time?

**Figure 3:**
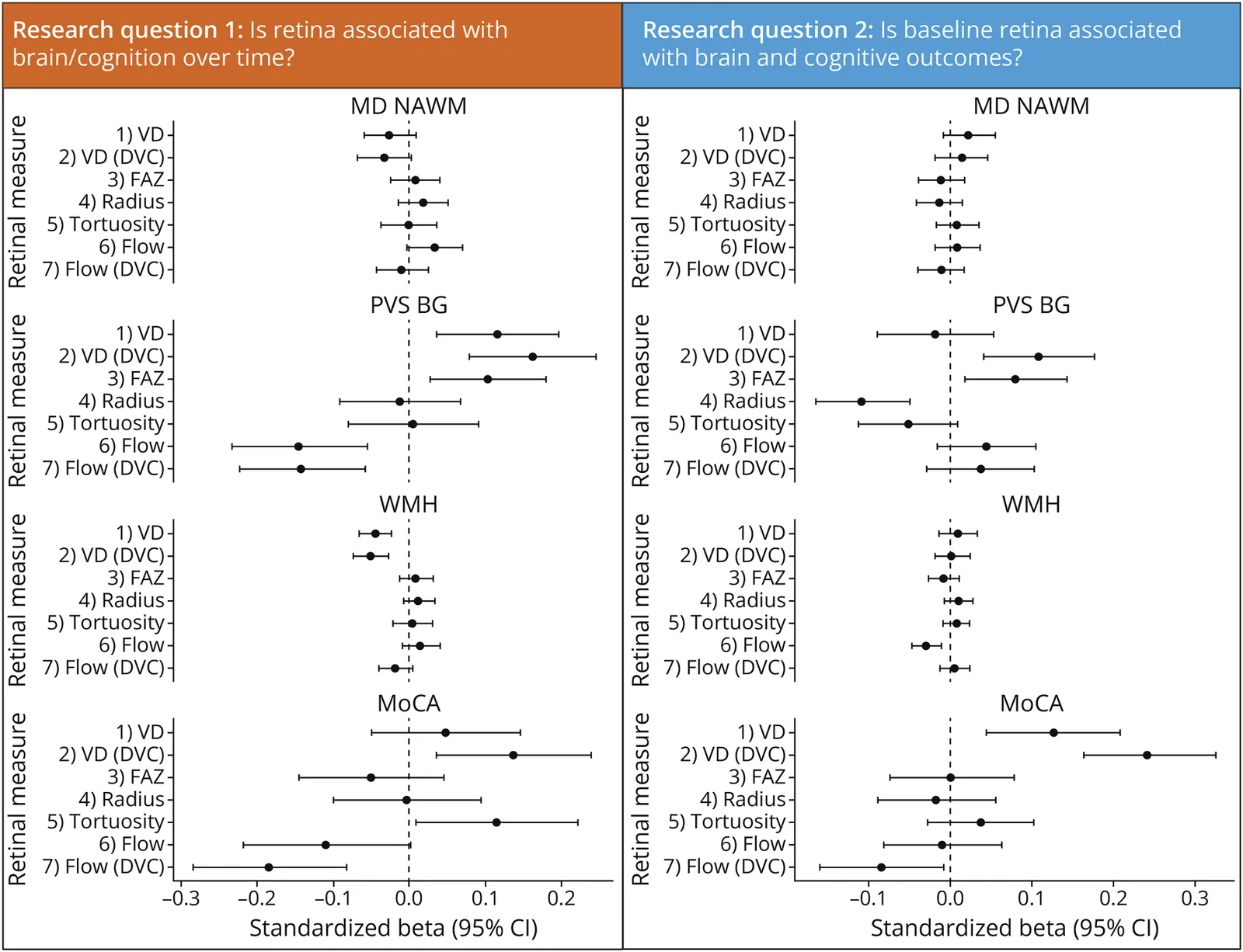
Forest Plots Representing Fully Adjusted Results for Both Primary Research Questions All models adjusted for age, sex, systolic blood pressure, smoking, diabetes, NIHSS, modified Rankin Score, and stroke type. DVC = deep vascular complex; FAZ = foveal avascular zone; MD NAWM = mean diffusivity in normal appearing white matter; MoCA = Montreal Cognitive Assessment; NIHSS = NIH Stroke Scale; PVS BG = perivascular spaces in basal ganglia; VD = vascular density; WMHs = white matter hyperintensities.

We first asked whether retinal microvascular changes tracked with brain structural and cognitive changes over time. After adjusting for covariates, increases in retinal vascular density were associated with increases in PVS volume (standardized β [95% CI] superficial layer 0.115 [0.035–0.195]; deep layer 0.162 [0.080–0.244]), reductions in WMH volume (superficial layer −0.045 [–0.067 to −0.023]; deep layer −0.050 [–0.074 to −0.026]), and conversely, decreases in retinal vascular density were associated with worsening MoCA (deep layer 0.136 [0.036, 0.236]). Increases in retinal FAZ area were associated with increases in PVS volume (0.102 [0.025, 0.179]). Decreases in retinal vascular tortuosity were associated with worsening MoCA (0.114 [0.008, 0.220]). Finally, increases in retinal flow were associated with reductions in PVS volume (superficial −0.144 [–0.232 to −0.056]; deep −0.143 [–0.226 to −0.060]). No significant associations were observed for mean diffusivity. Forest plots for fully adjusted models are presented in Figure 3 (left panel). Univariate and adjusted models showed nearly identical results, indicating minimal confounding (eTable 1).

### Research Question 2: Is Baseline Retina Associated With Brain and Cognitive Outcomes?

Next, we asked whether baseline retinal measurements were associated with future brain structural and cognitive changes. After adjusting for covariates, increased retinal vascular density at baseline was associated with increased PVS volume at 1 year (0.107 for deep layer [0.040–0.174]), with conversely, reduced vascular density at baseline associated with worse MoCA at 1 year (for superficial layer 0.125 [0.043, 0.207]; for deep layer 0.242 [0.162–0.322]). Increased baseline retinal FAZ area was associated with increased PVS volume at 1 year (0.079 [0.016–0.142]). Finally, increased baseline flow in the deep retinal layer was associated with worse MoCA at 1 year (−0.083 [–0.159 to −0.007]), while increased flow in the superficial layer was associated with reduced WMH volume at 1 year (−0.029 [–0.047 to −0.011]). None of the baseline retinal measures were associated with change in mean diffusivity after adjustment. Forest plots for fully adjusted models are presented in Figure 3 (right panel). The full results for both univariate and fully adjusted models are presented in eTable 2.

## Discussion

This study provides evidence that longitudinal changes in retinal OCTA microvascular measurements are associated with progression of cSVD features and cognitive function over 1 year in a cohort of mild stroke patients enriched for cSVD. Our findings demonstrate that retinal OCTA measures are not only associated with brain cSVD over time but also also associated with future cSVD burden and lower cognitive scores at follow-up, suggesting the potential utility of OCTA as a noninvasive biomarker for cSVD prognosis and monitoring of disease progression.

The most robust finding was the inverse relationship between retinal vascular density and WMH progression, with greater reductions in retinal VD across both the superficial and deep layers associated with increasing WMH volume over time. This relationship was particularly pronounced in the deep vascular complex (DVC), which may reflect its closer anatomical correspondence to the deep penetrating arterioles affected in cSVD pathophysiology.^3^ Specifically, a 1 SD decrease in deep VD was associated with a β = −0.050 SD increase in WMH volume over 1 year, estimated at ∼0.29 mL. (Calculated with back-transformation, which is approximate, because it assumes a gamma-distributed WMH variable based on the reported mean and SD in Table 1 and a mean ICV of ∼1,600 mL across visits.) For context, 0.25 mL/y represents the threshold of reliably detectable WMH change on MRI.^34^ Notably, the annualized WMH progression benefit in the intensive blood pressure lowering arm of SPRINT-MIND (Systolic Blood Pressure Intervention Trial-Memory and Cognition in Decreased Hypertension) was approximately 0.14 mL/y,^35^ less than our estimated effect, suggesting that the association reported here is not negligible in clinical terms. Notably, neither superficial nor DVC VD predicted net WMH volume at 1 year. However, this baseline to 1-year analysis does not account for the dynamic nature of WMH, which can both increase and decrease over time—a factor captured by examining these associations across visits. The deep retinal capillary plexus shares similar structural characteristics with cerebral penetrating vessels and surrounding membranes, including comparable vessel caliber, branching patterns, and susceptibility to similar vascular risk factors.^5^ Our results suggest that retinal microvascular rarefaction may parallel the progressive intrinsic disease of cerebral small vessels that underlies WMH development and progression.

The associations between retinal parameters and PVS changes suggested a more complex pattern. Increasing PVS volume was associated with higher retinal vascular density and reduced blood flow, particularly within the DVC. Although increased PVS volume is widely considered a marker of cerebral small vessel dysfunction,^36^ it is not specific to a single underlying pathologic process and may reflect a range of processes or mechanisms, including altered interstitial fluid dynamics or inflammatory processes. The association between higher retinal vascular density and greater PVS volume alongside reduced WMH volume may seem counterintuitive but likely reflects the temporal dissociation between these cSVD markers. PVS enlargement is thought to represent an earlier, more dynamic alteration in perivascular fluid clearance (i.e., an early stage that could lead to tissue damage or resolve without lasting injury),^37^ whereas WMH reflect more overt tissue injury, ranging from potentially reversible^31,32^ to established damage. Indeed, increasing evidence shows that WMHs form around dilated PVS.^38,39^ Relatively preserved microvascular integrity, indexed by higher retinal vascular density, may coexist with PVS enlargement in earlier disease stages, while preceding the tissue damage that manifests as WMH.

Notably, higher retinal vascular density in this context does not necessarily imply an increased number of vessels but may instead reflect changes in vessel caliber or morphology that increase the proportion of perfused tissue detected on OCTA.

Together, these findings are consistent with the possibility that, at an early stage of microvascular dysfunction, potentially preceding overt WMH formation or worsening,^40^ vascular structural or functional alterations occur before the vessel rarefaction typically observed in more advanced cSVD.^41^

Our baseline-adjusted models (RQ2) revealed that baseline retinal OCTA parameters are associated with brain and cognitive outcomes, supporting their potential role in risk stratification. Lower baseline retinal vascular density in both layers was strongly associated with lower cognitive scores at 1 year. Specifically, a 1 SD unit decrease in deep VD at baseline was associated with a 0.83-point decrease in MoCA over 1 year (0.242 in standardized units). For context, the minimal clinically important difference in MoCA has been estimated as approximately 1 point in a large study of stroke survivors,^42^ suggesting that while the observed association is statistically robust, it is modest in absolute terms and should be interpreted as reflecting subclinical variation across the population rather than individual-level clinical change. The standardized β of 0.242 represents a small-to-moderate effect size and is consistent with similar work in this cohort.^31^ This finding aligns with previous cross-sectional reports showing associations between lower VD and worse cognitive function.^19,23,24,26^ However, our findings contrast with the large (N = 967) population-based Eye Determinants of Cognition (EyeDOC) study,^43^ which found no associations between OCTA VD and longitudinal cognitive change or incident mild cognitive impairment/dementia in community-dwelling older adults. This discrepancy could be explained by population differences: Our cohort comprised patients (mean age around 65 years) with recent stroke and relatively preserved cognition who demonstrated cognitive improvement over follow-up (Table 1). By contrast, the EyeDOC study examined older adults (mean age around 79 years) experiencing typical age-related cognitive decline. The utility of baseline retinal measures for later outcomes may therefore be most apparent in populations with active cerebrovascular pathophysiology, suggesting that the clinical application of retinal biomarkers may require careful consideration of patient and disease context.

Higher baseline retinal OCTA flow was weakly associated with reduced WMH progression over 1 year, suggesting that preserved or improved retinal perfusion may reflect improved cerebral perfusion and be protective against white matter deterioration or even facilitate WMH reduction. This finding supports the idea that adequate microvascular perfusion is essential for maintaining tissue integrity because better baseline retinal blood flow likely reflects healthier small vessel function that can support metabolic demands and prevent ischemic white matter damage.^3,41,44^ The protective association between retinal OCTA perfusion and future white matter outcomes reinforces the potential utility of retinal flow measures for identifying individuals with preserved microvascular reserve who may be more resilient to cerebrovascular progression.

The association between higher deep retinal layer flow and worse cognition (RQ1 and RQ2) may reflect compensatory hyperperfusion in early microvascular dysfunction, representing autoregulatory strain rather than vascular health. However, this is speculative and warrants further investigation.

In previous work, we reported cross-sectional associations between diffusion MRI and OCTA measures^27^ and longitudinal associations between diffusion measures and cognition.^45^ This discrepancy may reflect differences in sample size or OCTA measurement software. Notably, VD measures approached significance in RQ1, suggesting that, such as WMH, mean diffusivity (MD) changes may be bidirectional. This bidirectionality could obscure associations with net MD change while still revealing trends when examining concurrent changes in VD and MD, similar to the pattern observed with WMH.

These findings have several clinical implications. First, the ability to monitor associations between retinal and brain microvasculature noninvasively over time could facilitate mechanistic understanding as well as monitoring of therapeutic interventions in clinical trials, potentially serving as a more accessible and cost-effective end point than repeated brain MRI,^46^ although obtaining high enough quality OCTA for accurate readings can be challenging.^43,47^ Second, they support the development of retinal OCTA as a screening tool for individuals at risk of cSVD, potentially enabling earlier detection of microvascular dysfunction before overt brain lesions develop. Finally, the baseline retina ∼ future brain/cognition relationships suggest that retinal measures could be used for risk stratification, helping identify patients who may benefit from more aggressive vascular risk factor modification.

Future research should focus on several key areas. Longer follow-up periods would help determine whether the relationships we observed persist over time and whether retinal changes precede or follow brain/cognition changes. Investigation of the optimal OCTA parameters and imaging protocols for cSVD monitoring would help standardize approaches across research centers. In addition, validation of these findings in larger, more diverse populations would strengthen the generalizability of retinal biomarkers for cerebrovascular disease. Stratification by putative cSVD subtype (e.g., cerebral amyloid angiopathy (CAA), CADASIL, and hypertensive arteriopathy) was not part of the study protocol. Based on imaging characteristics such as microbleed locations and related diagnostic features for CAA,^48^ very few participants were likely to have had CAA. However, future work should investigate brain-retina coupling across different cSVD subtypes because retinal-brain associations may differ across these distinct entities. Establishing minimal detectable change thresholds for OCTA-derived retinal vascular measures across a broader range of patient populations remains an important priority for future work. Finally, further analysis could explore the effects of interocular OCTA asymmetry on MRI and cognitive measures in this cohort.^49^

This study has several strengths, including its longitudinal design in a well-characterized cohort, comprehensive retinal and brain imaging protocols, good retention, and the use of validated, quantitative analysis methods and quality checking. The OCTARIMA software provides standardized, reproducible metrics that can be implemented across different research centers. The mixed-effects modelling approach appropriately accounts for repeated measures and missing data while controlling for relevant confounders.

However, several limitations warrant consideration. The 1-year follow-up period, while sufficient to detect meaningful changes, may not capture the full temporal sequence of retinal-brain/cognition relationships. The study population consisted of mild stroke patients, which may limit generalizability to asymptomatic individuals or those with more severe cerebrovascular disease. Quality control led to exclusion of some OCTA scans, potentially introducing selection bias toward participants with better image quality and reducing statistical power. We did not assess test-retest reproducibility of OCTA metrics within this cohort. However, OCTARIMA has demonstrated excellent reproducibility in prior work.^50^ Future work should establish the minimal detectable change for these metrics in stroke populations specifically to determine whether longitudinal changes observed here exceed measurement noise. We did not adjust for atrial fibrillation; however, its prevalence was low (approx. 9.2%). The effect sizes reported here, while statistically significant, are modest in absolute terms; their clinical significance in individual patients remains to be established. Finally, the exploratory nature of our analysis, without correction for multiple comparisons, requires cautious interpretation of individual findings, although the overall pattern of results supports meaningful retinal brain/cognition relationships.

This study provides evidence that longitudinal retinal microvascular measures are associated with cSVD progression and that baseline OCTA is associated with future brain and cognitive outcomes. The ability of OCTA to track these relationships noninvasively supports its potential as a biomarker for cSVD monitoring and risk stratification. These findings advance our understanding of retinal-brain microvascular coupling and support continued development of retinal imaging for cerebrovascular health assessment. However, given the heterogeneous and enriched nature of the cohort, these associations cannot be attributed specifically to cSVD pathophysiology and should be interpreted as hypothesis generating. Future research should focus on validating these relationships over longer periods and in broader populations to fully realize the clinical potential of retinal biomarkers in cerebrovascular medicine.

## Glossary

cSVD: cerebral small vessel disease
DVC: deep vascular complex
EyeDOC: Eye Determinants of Cognition
FAZ: foveal avascular zone
ICV: intracranial volume
MoCA: medical and cognitive assessment
mRS: modified Rankin Scale
MSS3: Mild Stroke study 3
OCTA: optical coherence tomography angiography
PVS: perivascular space
VD: vessel density
WMH: white matter hyperintensity
